# Perceptions of Patients With Stroke Regarding an Immersive Virtual Reality–Based Exercise System for Upper Limb Rehabilitation: Questionnaire and Interview Study

**DOI:** 10.2196/49847

**Published:** 2025-01-01

**Authors:** Jiayin Chen, Calvin Kalun Or, Zhixian Li, Eric Hiu Kwong Yeung, Tianrong Chen

**Affiliations:** 1 Department of Ergonomics and Healthcare College of Furniture and Industrial Design Nanjing Forestry University Nanjing China; 2 Department of Data and Systems Engineering University of Hong Kong Hong Kong China (Hong Kong); 3 Department of Rehabilitation Medicine Dingzhou People’s Hospital Dingzhou China; 4 The University of Hong Kong–Shenzhen Hospital Department of Physiotherapy The University of Hong Kong–Shenzhen Hospital Shenzhen China

**Keywords:** virtual reality, stroke, perception, rehabilitation, questionnaire, interview

## Abstract

**Background:**

With substantial resources allocated to develop virtual reality (VR)–based rehabilitation exercise programs for poststroke motor rehabilitation, it is important to understand how patients with stroke perceive these technology-driven approaches, as their perceptions can determine acceptance and adherence.

**Objective:**

This study aimed to examine the perceptions of patients with stroke regarding an immersive VR-based exercise system developed to deliver shoulder, elbow, forearm, wrist, and reaching exercises.

**Methods:**

A questionnaire was used to assess the perceptions of 21 inpatients who had experienced stroke (mean time from stroke onset: 37.2, SD 25.9 days; Brunnstrom stage of stroke recovery for the arm: 3-5) regarding the perceived usefulness of, ease of use of, attitude toward, intrinsic motivation for, and intention to use the exercise system. The measurement items were rated on a 7-point Likert scale ranging from 1 (very strongly disagree) to 7 (very strongly agree), with higher values indicating more positive perceptions. Descriptive statistics were used to summarize the responses. Moreover, we conducted semistructured interviews that were audio recorded, transcribed, and subjected to content analysis to identify thematic patterns.

**Results:**

The questionnaire results revealed that the patients’ perceptions of the exercise system were positive (mean ratings >6). The content analysis revealed 6 positive themes from 73 statements about the exercise system: ease of use, usefulness, enjoyment, motivation, accessibility, and game design. Conversely, 15 statements reflected negative perceptions, which were clustered into 3 themes: difficulty in handling VR devices, uncomfortable experiences when using VR devices, and monotony.

**Conclusions:**

Integrating VR technology into poststroke functional exercises holds significant promise based on patient interests. However, patient preferences and adaptability must be considered to promote the technology’s success. VR-guided exercises should be user-friendly, health-promoting, engaging, and well-designed. Furthermore, addressing challenges, such as bulkiness, motion sickness, discomfort, and exercise monotony, is crucial for the widespread adoption and diffusion of this technology.

## Introduction

Persistent upper limb impairment following stroke has a substantial impact on patients’ daily activities and quality of life [1-5]. Therapeutic exercises are necessary to restore motor function and independence. Patients commonly attend face-to-face therapy sessions at clinics and perform exercises under the supervision of therapists. However, this traditional approach has limitations, including high costs, scheduling conflicts, limited access to therapy services, and the need for patients to travel to clinics [6,7].

Technologies, such as immersive virtual reality (VR), have been applied to promote the accessibility and affordability of poststroke therapeutic exercises [8-10]. The effectiveness of immersive VR-based rehabilitation programs on upper limb motor recovery has been examined in previous studies [11-13]. Besides effectiveness, it is equally important to consider patients’ perceptions and acceptance of such programs because negative perceptions and nonacceptance of these technologies, which is a common challenge in practice, can lead to implementation failure, losses to stakeholders, and undesirable health care outcomes [14,15].

Several studies have examined the experiences of stroke survivors with immersive VR-based motor rehabilitation programs, evaluating various aspects such as perceived usefulness, discomfort, motivation, and intention to use these programs. Results have shown that the application of immersive VR is both feasible and acceptable among patients [13,16-18]. However, these studies primarily relied on quantitative methods, specifically rating scales, to capture the experiences of patients. This approach, while valuable, fails to uncover the underlying factors that influence these experiences, leaving critical elements unexplored [19]. To address this gap, incorporating qualitative methods, such as in-depth interviews, is essential. Qualitative research can provide rich, detailed data that reveals the complexities of patients’ interactions with VR technology, their emotional responses, and the contextual factors that shape their experiences. By exploring these dimensions, researchers can identify barriers and facilitators to the formation of positive perceptions and subsequent acceptance and implementation. This will allow them to tailor interventions to meet the specific needs of patients with stroke and enhance the overall effectiveness of VR-based rehabilitation programs.

Currently, qualitative studies evaluating the experiences of stroke survivors with immersive VR-based rehabilitation programs are limited. Previous studies with small sample sizes have provided some insights but are insufficient to understand broader experiences and perceptions of such patients [20,21]. Therefore, this study aimed to conduct both quantitative and qualitative analyses of the perception of patients with stroke regarding an immersive VR-based exercise system for poststroke upper limb exercises. Through this mixed methods approach, we aim to obtain a better understanding of the factors influencing perceptions and identify areas for improvement in the design and implementation of VR-based rehabilitation programs.

## Methods

### Data Source

**Figure 1:**
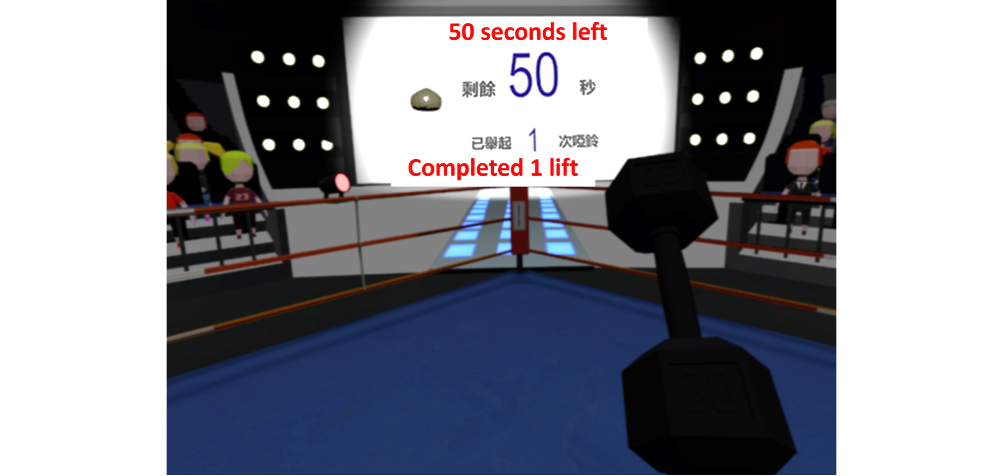
Dumbbell-lifting game for shoulder exercise.

**Figure 2:**
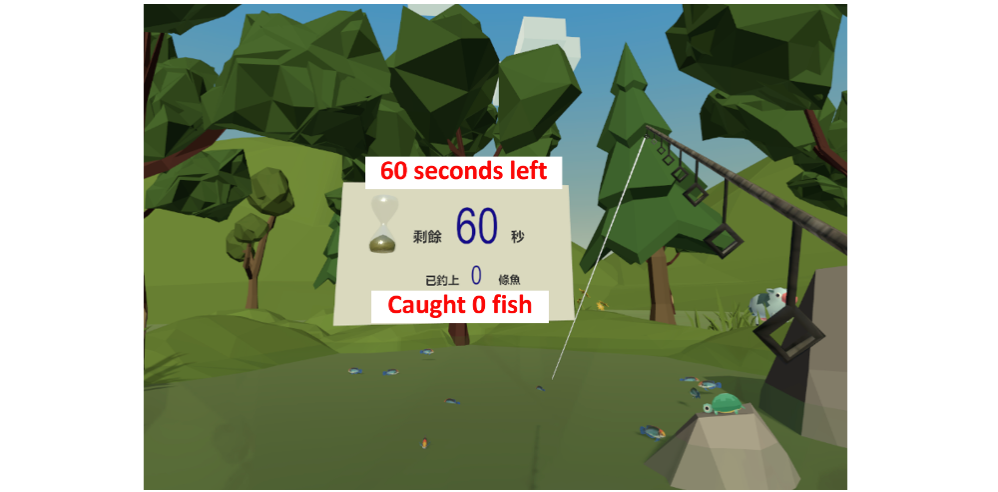
Fishing game for elbow exercise.

**Figure 3:**
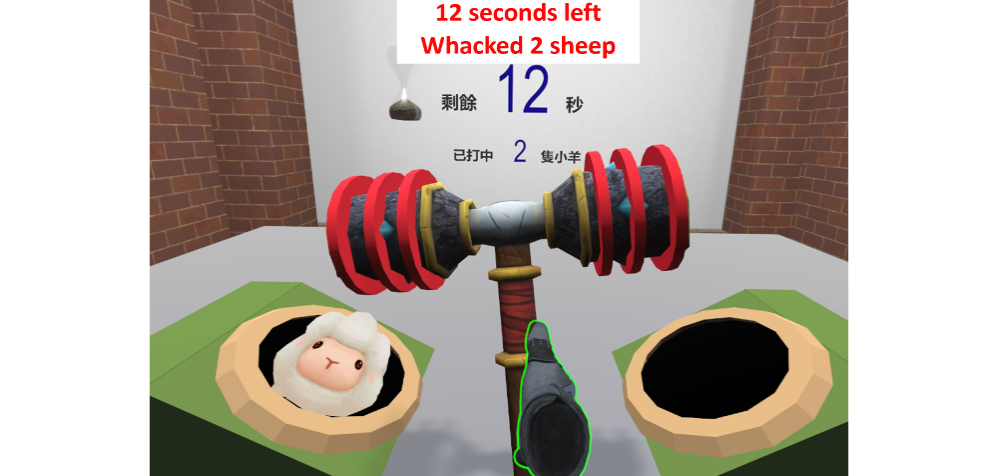
Sheep-whacking game for forearm exercise.

**Figure 4:**
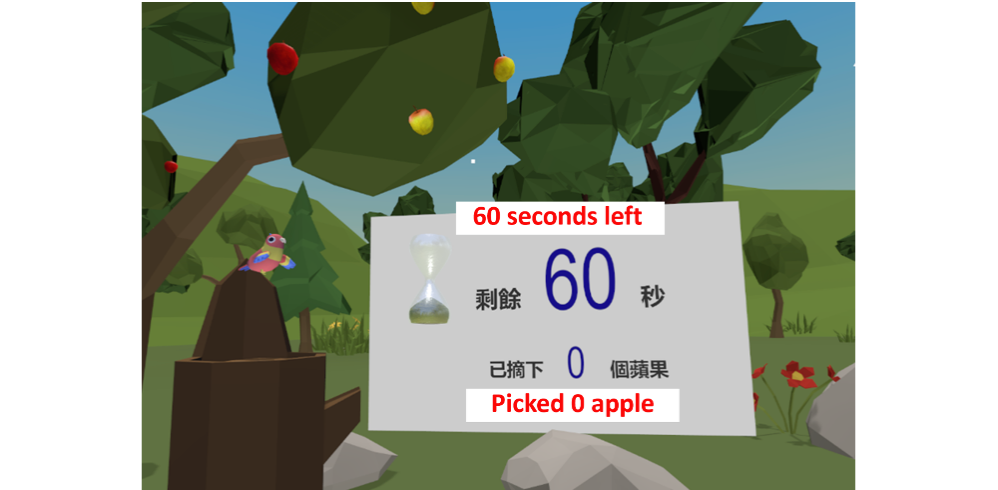
Apple-picking game for wrist exercise.

**Figure 5:**
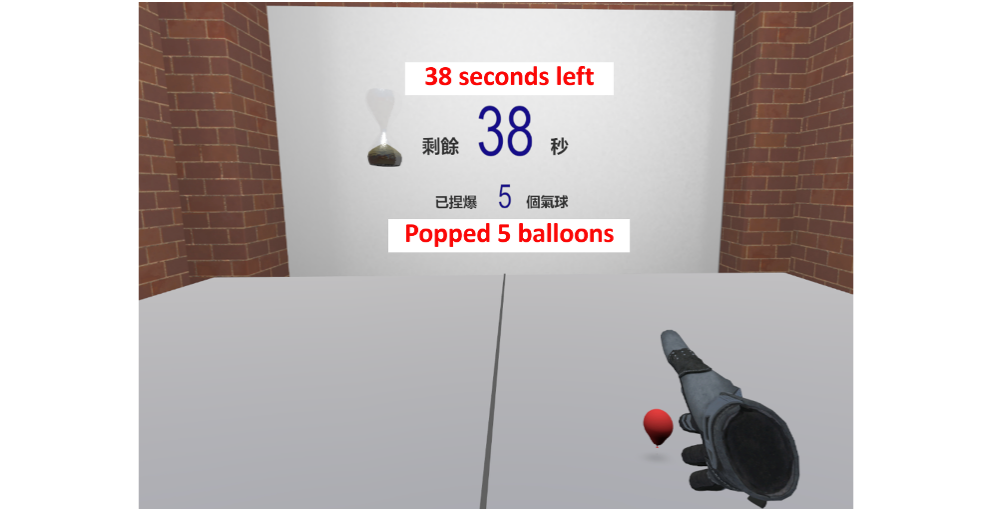
Balloon-popping game for reaching exercise.

**Figure 6:**
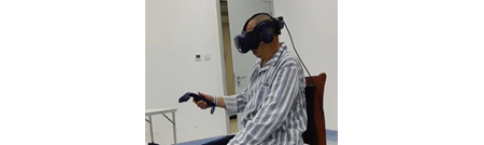
A participant performing exercises using the virtual reality device.

The data source was a questionnaire survey and semistructured interviews conducted as part of a proof-of-concept randomized controlled trial (RCT) [11]. The trial examined the effectiveness and safety of an immersive VR-based exercise system for poststroke upper limb exercises. The exercise system comprised 5 games (Figures 1-5): dumbbell-lifting game for shoulder flexion and abduction, fishing game for elbow flexion, sheep-whacking game for forearm pronation and supination, apple-picking game for wrist flexion and extension, and balloon-popping game for reaching exercises. The details of the 5 games are presented in Multimedia Appendix 1. A VR headset and a wireless handheld controller (HTC VIVE Pro, HTC Corporation) were used to display and interact with the virtual environments (Figure 6).

Study participants were inpatients at a public hospital in China who have had a stroke. Patients were considered eligible for the study if they were aged 19 to 75 years; had their first ever unilateral stroke, as confirmed by their computed tomography or magnetic resonance imaging records; were experiencing motor impairments in one of the upper limbs, with Brunnstrom stages of stroke recovery of the arm of 3 to 5; had an active range of motion of at least 10° in the shoulder and elbow of the affected arm; could maintain autonomous upright seating for at least 45 minutes; and had normal or corrected-to-normal vision and hearing. Patients with injuries or other health conditions restricting their upper limb mobility, unilateral spatial neglect, unstable medical conditions, a history of seizures or epilepsy, communication difficulty, mood instability, or concurrent participation in any other ongoing investigational drug studies were excluded. Physicians and physical therapists in the rehabilitation medicine department initially introduced the study to their inpatients. Patients expressing interest in participation were invited to a screening session, during which they tried the study’s VR devices, and their eligibility was assessed. The researcher (JC) provided detailed explanations of the study to the patients initially determined eligible, confirmed their eligibility, and obtained their written informed consent for participation. In the RCT, participants were randomly assigned to one of the 2 study groups. The intervention group received the aforementioned exercise system and was instructed to use the system to perform upper limb exercises for 35 minutes each day, 6 days a week, for 2 weeks. The control group received commercial games downloaded from Steam (Valve Corporation) and was instructed to play them for the same duration and frequency [22]. These commercial games served as a sham VR program to conceal group assignments from participants. They were not directly aligned with the exercises provided in the exercise system and were used solely for entertainment. Multimedia Appendix 2 provides details of these commercial games. The control group used the same headset and handheld controller as the intervention group. All participants continued their prescribed stroke rehabilitation therapy in the hospital throughout the study period.

The participants’ demographic and clinical characteristics, including age, sex, education level, stroke type, time from stroke onset, Brunnstrom stage of the affected arm, and previous experience with VR, were collected at baseline. The effectiveness and safety of the immersive VR-based exercise system were measured at baseline and the 1-week and 2-week follow-up assessments. Owing to the exclusive use of the exercise system by the intervention group, perceptions of the exercise system were only evaluated in this group. A questionnaire survey and semistructured interviews were conducted during the participants’ last follow-up assessments; the details are provided in the Questionnaire and Interview sections.

### Ethics Approval

The RCT was approved by The University of Hong Kong–Shenzhen Hospital Human Research Ethics Committee (approval number [2021]137) and Dingzhou People’s Hospital (approval number hx-2021-04) in China. It has been registered in the Chinese Clinical Trial Registry (registration number ChiCTR2100047150). All the participants provided written informed consent before participating in the RCT. No cash compensation was provided. All data used were deidentified.

### Questionnaire

A questionnaire was adapted from technology acceptance models and relevant studies examining individuals’ and patients’ perceptions of various technologies [23-28]. It was used to assess participants’ perceptions of the exercise system, including perceived usefulness, perceived ease of use, attitude, intrinsic motivation, and behavioral intention. Textbox 1 presents the measurement items. The items were rated on a 7-point Likert scale ranging from 1 (very strongly disagree) to 7 (very strongly agree), with higher values indicating more positive perceptions.

**Perception outcomes and measurement items.**

**Perceived usefulness (PU)**

- PU 1: using the immersive virtual reality (VR)–based exercise system to perform upper limb exercises would improve upper limb motor function.
- PU 2: using the immersive VR-based exercise system to perform upper limb exercises would increase exercise efficiency.
- PU 3: using the immersive VR-based exercise system to perform upper limb exercises would enhance exercise effectiveness.
- PU 4: you found the immersive VR-based exercise system useful for performing upper limb exercises.

**Perceived ease of use (PEOU)**

- PEOU 1: learning to use the immersive VR-based exercise system was easy for you.
- PEOU 2: you found it easy to get the immersive VR-based exercise system to do what you wanted to do.
- PEOU 3: it was easy to become skillful at using the immersive VR-based exercise system.
- PEOU 4: you found the immersive VR-based exercise system easy to use.

**Attitude (ATT)**

- ATT 1: using the immersive VR-based exercise system to perform upper limb exercises is a good idea.
- ATT 2: using the immersive VR-based exercise system to perform upper limb exercises is a wise idea.
- ATT 3: you like the idea of using the immersive VR-based exercise system to perform upper limb exercises.
- ATT 4: using the immersive VR-based exercise system to perform upper limb exercises was a pleasant experience.

**Intrinsic motivation (IM)**

- IM 1: you found using the immersive VR-based exercise system to perform upper limb exercises enjoyable.
- IM 2: the actual process of using the immersive VR-based exercise system to perform upper limb exercises was pleasant.
- IM 3: you had fun using the immersive VR-based exercise system to perform upper limb exercises.

**Behavioral intention (BI)**

- BI 1: you intend to continue using the immersive VR-based exercise system to perform upper limb exercises.
- BI 2: You plan to continue using the immersive VR-based exercise system to perform upper limb exercises.

### Interview

As the RCT was a proof-of-concept trial, individual, face-to-face, and semistructured interviews were conducted to learn more about the participants’ experiences with the exercise system, allowing us to improve it further. Open-ended questions were asked regarding the exercise system’s comfort, comprehensibility, usefulness, and the participants’ preferences and intention to use the system. Textbox 2 presents the details of the interview questions. During the interview, a researcher (JC) reformulated and clarified questions to ensure the participants’ complete comprehension. Probing questions, such as “What do you mean by that?” and “Could you be more specific?” were also asked to obtain deeper insights into the participants’ responses when necessary.

**Interview questions.**

**Comfort**

- Do you feel any discomfort when using the immersive virtual reality (VR)–based exercise system?
- Do virtual environments make you feel uncomfortable?
- Does the headset or the handheld controller make you feel uncomfortable?
- (Ask if the participants reported discomfort) What makes you feel uncomfortable?

**Comprehensibility**

- Is it easy for you to understand the exercise tasks?
- Is it easy for you to use the headset or the handheld controller?
- (Ask if the participants reported difficulties) What do you find difficult to understand or use?

**Usefulness**

- Do you think that the immersive VR-based exercise system is useful for your upper limb rehabilitation?
- What makes you think the immersive VR-based exercise system is useful for your rehabilitation?
- What makes you think that the immersive VR-based exercise system is useless for your rehabilitation?

**Preference**

- Do you like the immersive VR-based exercise system?
- What aspects of the immersive VR-based exercise system do you like?
- What aspects of the immersive VR-based exercise system do you dislike?

**Intention to use**

- Do you intend to use the immersive VR-based exercise system for rehabilitation in the future?
- What motivates you to use the immersive VR-based exercise system for rehabilitation?
- What fails to make you intend to use the immersive VR-based exercise system for rehabilitation?

### Procedures

Written informed consent was obtained from each participant before enrollment into the RCT. Before starting, the researcher (JC) explained the objectives of the questionnaire and interview. The participants then completed the questionnaire to indicate their perceptions of the exercise system. Next, the researcher interviewed the participants. Verbal consent for audio recording was verified again before the start of the interviews. During the interviews, the participants were encouraged to express their experience with the exercise system as much as possible. The interview was audio recorded for subsequent transcription. The interviews were conducted in Chinese. The administration of the questionnaire and interview lasted approximately 20 minutes for each participant.

### Data Analysis

#### Questionnaire

Descriptive statistics, including the mean, SD, median, and rating distribution of the measurement items, were calculated.

#### Interview

The interview audiotapes were transcribed verbatim by the researcher (JC). Two researchers (JC and TC) with backgrounds in human factors and health care informatics were involved in the analysis. The interview transcripts were analyzed using a qualitative content analysis approach with 3 steps [29]. Step 1: the interview transcripts were read several times before being independently analyzed by the 2 researchers. During the initial reading, the 2 researchers read through the interview transcripts while listening to the interview audiotapes to verify the accuracy of the transcripts and to gain an overall sense of the interview content. During the subsequent reading, the 2 researchers read through the interview transcripts in detail to gain a thorough understanding of the interview content. Step 2: an inductive approach was used to analyze the data because this approach allows the identification of codes and themes that answer the research question [30]. During the data analysis process, the first interview transcript was independently analyzed by the 2 researchers. Relevant statements from the interview transcript were identified, and codes were generated, which were then refined and developed into themes. The same process was repeated for each interview transcript, with the codes and themes added, revised, or developed each time. In addition, the frequencies of the statements expressed by the participants were recorded. Step 3: after all the interview transcripts were analyzed, the codes and themes were compared between the 2 researchers. In case of discrepancies, the original interview transcripts were checked, and the discrepancies were resolved through discussion between the 2 researchers until a consensus was reached.

NVivo (version 12; Lumivero) was used for content analysis, which was based on a checklist of consolidated criteria for reporting qualitative research [31].

## Results

### Participant Characteristics

Of the 25 intervention group participants, 3 were discharged from the hospital in advance and 1 withdrew from the study, leaving 21 participants who completed the questionnaire and interviews. The demographic and clinical characteristics of the 21 participants are presented in Table 1. The mean age was 55.3 (SD 12.9) years, and the participants were predominantly male (15/21, 71%). Two-thirds of the participants had completed their secondary school education (14/21, 67%). Two-thirds of the participants had an ischemic stroke (14/21, 67%), with a mean time from stroke onset until study enrollment of 37.2 (SD 25.9) days. More than half of the participants (13/21, 62%) were at Brunnstrom stage 3. Most participants had no experience using a VR device (20/21, 95%).

**Table 1:** Characteristics of the participants (N=21).

Characteristics			Values
**Age (y)**			
	Mean (SD)	55.3 (12.9)	
	Median (range; IQR)	54 (28-75; 46-66.5)	
**Sex, n (%)**			
	Male	15 (71)	
	Female	6 (29)	
**Education level, n (%)**			
	No schooling completed	0 (0)	
	Some level of primary schooling	0 (0)	
	Primary school completed	6 (29)	
	Some level of secondary schooling	0 (0)	
	Secondary school completed	14 (67)	
	Diploma, advanced diploma, associate degree, or equivalent	1 (5)	
	Bachelor’s degree	0 (0)	
**Stroke type, n (%)**			
	Ischemic	14 (67)	
	Hemorrhagic	7 (33)	
**Time from stroke onset at enrollment (d)**			
	Mean (SD)	37.2 (25.9)	
	Median (range; IQR)	34 (7-107; 18-56)	
**Brunnstrom stage of the affected arm, n (%)**			
	3	13 (62)	
	4	4 (19)	
	5	4 (19)	
**Experience using a VR^a^** **device, n (%)**			
	Never	20 (95)	
	Rarely	1 (5)	
	Sometimes	0 (0)	
	Often	0 (0)	
	Always	0 (0)	

^a^VR: virtual reality.

### Questionnaire

Table 2 presents the distribution, mean (SD), and median of the participants’ ratings for each measurement item on the questionnaire. The mean values of the overall ratings for perceived usefulness, perceived ease of use, attitude, intrinsic motivation, and behavioral intention were 6.20 (SD 0.69), 6.33 (SD 0.80), 6.25 (SD 0.74), 6.37 (SD 0.75), and 6.31 (SD 0.84), respectively. The median ratings for each measurement item were 6 or 7.

**Table 2:** Distribution, mean (SD), and median of the participant’s responses to the measurement items (N=21).

Outcomes		Rating distribution, n (%)								Mean (SD)		Median
		1	2	3	4	5	6	7				
**PU^a^**												
	PU 1	0 (0)	0 (0)	0 (0)	0 (0)	5 (24)	7 (33)	9 (43)	6.19 (0.81)		6	
	PU 2	0 (0)	0 (0)	0 (0)	0 (0)	5 (24)	8 (38)	8 (38)	6.14 (0.79)		6	
	PU 3	0 (0)	0 (0)	0 (0)	0 (0)	4 (19)	10 (48)	7 (33)	6.14 (0.73)		6	
	PU 4	0 (0)	0 (0)	0 (0)	0 (0)	2 (10)	10 (48)	9 (43)	6.33 (0.66)		6	
**PEOU^b^**												
	PEOU 1	0 (0)	0 (0)	0 (0)	0 (0)	3 (14)	6 (29)	12 (57)	6.43 (0.75)		7	
	PEOU 2	0 (0)	0 (0)	0 (0)	1 (5)	3 (14)	7 (33)	10 (48)	6.24 (0.89)		6	
	PEOU 3	0 (0)	0 (0)	0 (0)	1 (5)	2 (10)	7 (33)	11 (52)	6.33 (0.86)		7	
	PEOU 4	0 (0)	0 (0)	0 (0)	1 (5)	3 (14)	5 (24)	12 (57)	6.33 (0.91)		7	
**ATT^c^**												
	ATT 1	0 (0)	0 (0)	0 (0)	0 (0)	5 (24)	6 (29)	10 (48)	6.24 (0.83)		6	
	ATT 2	0 (0)	0 (0)	0 (0)	1 (5)	3 (14)	7 (33)	10 (48)	6.24 (0.89)		6	
	ATT 3	0 (0)	0 (0)	0 (0)	0 (0)	5 (24)	8 (38)	8 (38)	6.14 (0.79)		6	
	ATT 4	0 (0)	0 (0)	0 (0)	0 (0)	3 (14)	7 (33)	11 (52)	6.38 (0.74)		7	
**IM^d^**												
	IM 1	0 (0)	0 (0)	0 (0)	0 (0)	3 (14)	7 (33)	11 (52)	6.38 (0.74)		7	
	IM 2	0 (0)	0 (0)	0 (0)	0 (0)	4 (19)	6 (29)	11 (52)	6.33 (0.80)		7	
	IM 3	0 (0)	0 (0)	0 (0)	0 (0)	3 (14)	7 (33)	11 (52)	6.38 (0.74)		7	
**BI^e^**												
	BI 1	0 (0)	0 (0)	0 (0)	1 (5)	2 (10)	7 (33)	11 (52)	6.33 (0.86)		7	
	BI 2	0 (0)	0 (0)	0 (0)	1 (5)	2 (10)	8 (38)	10 (48)	6.29 (0.85)		6	

^a^PU: perceived usefulness.

^b^PEOU: perceived ease of use.

^c^ATT: attitude.

^d^IM: intrinsic motivation.

^e^BI: behavioral intention.

### Interviews

#### Overview

A total of 87.23 minutes of interview audiotapes were recorded. The interviews lasted between 2.43 and 8.30 (mean 4.15; median 3.88) minutes. The results of the content analysis were categorized into positive and negative perceptions of the exercise system. The details of the qualitative data are presented in the following sections.

#### Positive Perceptions of the Exercise System

##### Overview

A total of 73 statements derived from 21 participants were coded as positive perceptions. Of these, 25 (34%) statements were related to ease of use, 20 (27%) to benefits, 9 (12%) to enjoyment, 7 (10%) to assistance, 7 (10%) to accessibility, and 5 (7%) to game design. The findings are summarized in Table 3.

**Table 3:** Positive perceptions of the exercise system (N=21).

Themes, subthemes, and statements				Participants who made the statement, n
**Ease of use**				
	**Easy to understand games**			
		Easy to understand how to play the games	20	
	**Easy to use VR^a^** **devices**			
		Easy to use the head-mounted display and handheld controller	5	
**Benefits**				
	**Improved upper limb motor function**			
		Improved muscle strength	5	
		Improved range of motion	3	
		Improved stability	3	
		Improved overall movement performance	2	
		Improved flexibility	1	
	**Improved daily living skills**			
		Improved self-care skills in daily life	1	
	**Improved cognitive ability**			
		Improved reaction speed	2	
		Promoted brain thinking	1	
	**Improved mood**			
		Was in a good mood	2	
**Enjoyment**				
	**Had fun exercising**			
		Had fun during the exercise	9	
**Assistance**				
	**Assisted in motor learning**			
		Assisted the affected upper limb in developing normal movement patterns	7	
**Accessibility**				
	**Promoted access to exercise**			
		Provided more opportunities for exercises	7	
**Game design**				
	**Virtual environments**			
		Well-created virtual environments	2	
	**Feeling of immersion**			
		High immersion level	2	
	**Novelty**			
		Novel games	1	

^a^VR: virtual reality.

##### Ease of Use

Of the 21 participants, 20 expressed that the games were easy to understand. One of them felt that it was difficult to play the games the first time because of unfamiliarity, although it became easy after a few trials. A total of 5 participants reported that the headset and handheld controller were easy to use. Some participants stated the following:

[I] know how to play each [game].

Participant 20

It is difficult [to play the games] the first time when you are not familiar, but after you practice a few times, it is not difficult, because you get to know what is going on.

Participant 1

Picking up apples [the apple-picking game] is used to extend [the wrist]. I know it.

Participant 18

No difficulties [in using the headset or the handheld controller].

Participant 3

##### Benefits

Some participants reported that their upper limb motor function had improved. Specifically, 5 out of 21 participants expressed that their muscle strength had increased, 3 reported that their range of motion had improved, 3 expressed that their affected upper limb became more stable, 2 expressed that their overall upper limb movement performance had improved, and 1 said that their hand became more flexible. Some examples of the participants’ statements are as follows:

Muscle strength is a little better. There is no obvious [feeling] when putting the weight [on the wrist]. [It is] not tiring.

Participant 17

[I] can get the elbow straight.

Participant 13

Wasn’t it [the arm] wobbly before? The balance does not seem good. Now it [the arm] is not wobbly like that.

Participant 18

It [the immersive VR-based exercise system] makes your hands more flexible and softer.

Participant 1

The joints can move to the correct location.

Participant 7

It [the immersive VR-based exercise system] is good for arm movements, meaning your movements are more standard.

Participant 3

One participant observed improvements in their ability to perform self-care eating activities:

I can easily pick up and hold the food now. [I needed to] break it [into small pieces] before. I was afraid that [I] could not hold it and dropped it. Now I do not need to break it [into small pieces], I can hold it and eat it directly. I can also hold the cup when brushing [my] teeth and cleaning [my] mouth.

Participant 18

Two participants mentioned that playing games improved their reaction speed to the computer, and 1 participant expressed that playing games could promote thinking:

To the computer...I feel that [my] reaction is faster.

Participant 9

[The immersive VR-based exercise system] can facilitate your thinking and make the brain work hard.

Participant 1

Two participants expressed that they were in a good mood when immersed in the virtual environments.

I have been kind of stressed since I got this disease, but once I listen to that music, [my] mood turns good....I feel like I am alive again, walking into that wood and picking apples like [in] real [life], and being immersed into that environment. I feel like ... my mind broadens.

Participant 1

[I] feel like myself...anyway, I feel as if I am still useful, I will be able to do housework like before, to do this and that. It seems like the feeling of pessimism has been reduced.

Participant 1

##### Enjoyment

Of the 21 participants, 9 described their feelings of enjoyment and fun when performing VR exercises. For example, some participants stated as follows:

At least performing [exercises] with it [the immersive VR-based exercise system] is not that annoying, not that boring.

Participant 5

You are free to play this [game]. It is also exciting.

Participant 8

It [the immersive VR-based exercise system] is quite fun and can also train the function of the limbs.

Participant 19

##### Assistance

Of the 21 participants, 7 expressed that they might not know how to perform some movements in the real world; however, the virtual objects in the games guided and helped them to learn and encouraged them to try to perform the movements. Some participants stated as follows:

You know [I] could not do that movement [elbow extension] before. I feel it [the immersive VR-based exercise system] is useful. [The elbow] could extend when [I] put the fishing rod down.

Participant 5

[When] you would attempt to perform [the movements], it [the arm] could not move, but you would attempt to do it.

Participant 6

For example, that apple-picking [game], together with that bird, could guide my hand to perform the movements [wrist flexion and extension].

Participant 7

##### Accessibility

Of the 21 participants, 7 reported that the exercise system provided more opportunities for exercise. Some examples of the participants’ statements are as follows:

[I like using the immersive VR-based exercise system because I] can exercise. I can always exercise.

Participant 4

[I like using the immersive VR-based exercise system because it] provides arm exercises.

Participant 13

##### Game Design

Several participants expressed interest in the game design. Two out of 21 participants stated that the virtual environments were attractive, 2 stated that the games provided a sense of immersion, and 1 stated that the games were innovative. Some participants stated as follows:

[It is] just like being in a wonderland, right? It is also very clear, just like the 3D movies. I like looking around and performing the exercises.

Participant 21

There are images, music, and sounds of the birds. It makes you feel like you are in the woods. I feel very excited.

Participant 1

The games are novel.

Participant 17

#### Negative Perceptions of the Exercise System

##### Overview

A total of 15 statements derived from 8 participants were identified as negative perceptions. Of those, 8 (53%) statements were related to difficulty in handling VR devices, 6 (40%) statements to uncomfortable experiences when using VR devices, and 1 (7%) statement to monotony. The findings are summarized in Table 4.

**Table 4:** Negative perceptions of the exercise system (N=21).

Themes, subthemes, and statements				Participants who made the statement, n
**Difficulty in handling VR^a^** **devices**				
	**Bulky headset**			
		Heavy headset	3	
		Sweating and stuffiness when wearing the headset	3	
		Cable constraint	1	
	**Heavy handheld controller**			
		Heavy to hold handheld controller	1	
**Uncomfortable experiences when using VR devices**				
	**Dizziness**			
		Feeling dizzy when using the headset	2	
	**Fatigue**			
		Feeling tired during the exercise	2	
	**Eyestrain**			
		Eyestrain when using the headset	1	
	**Pain**			
		Pain during exercise	1	
**Monotony**				
	**Boring exercise**			
		Repetitive movements leading to monotony	1	

^a^VR: virtual reality.

##### Difficulty in Handling VR Devices

Of the 21 participants, 3 indicated that the headset was heavy, 3 mentioned that wearing the headset made them feel sweaty and stuffy, 1 stated that the cable connecting the headset and laptop constrained their head movement, and 1 mentioned that the handheld controller was heavy to hold. Some participants stated the following:

I feel that the headset is a little heavy.

Participant 5

It is a little sweaty [when wearing the headset]. It is not tiring; it is stuffy.

Participant 8

The head is being pressed. It is uncomfortable. [I] feel that it would be good if it were wireless [headset].

Participant 5

[If the headset is wireless], turning it [the head] around would be convenient and free.

Participant 5

It [the handheld controller] is just heavy. The total weight is heavy.

Participant 1

##### Uncomfortable Experiences When Using VR Devices

Two out of the 21 participants mentioned that wearing the headset led to dizziness, 2 reported that they experienced fatigue during the VR exercises, 1 highlighted that wearing the headset caused eyestrain, and 1 reported experiencing pain in the upper limb during the VR exercises. Some of the participants stated as follows:

[I] felt a little dizzy after taking off [the headset].

Participant 5

Watching [the virtual environments], the eyes are not very comfortable.

Participant 6

[The] part [of the upper limb that] worked hard...was sore.

Participant 7

It was a little painful when stretching.

Participant 18

##### Monotony

One participant reported that repeating the same exercises every day was boring, but the participant kept doing the exercises to recover faster.

For my own health, [I] mean to recover faster, the exercises must be performed, right? ...It is, of course, boring, always repeating the same movement.

Participant 18

## Discussion

### Principal Findings

#### Overview

This study aimed to investigate the perceptions of stroke survivors regarding the use of an immersive VR-based exercise system for poststroke upper limb exercises. Quantitative and qualitative analyses were used to explore these perceptions. The questionnaire results revealed that the participants recognized the exercise system’s usefulness for rehabilitation, found the exercise system easy to use, maintained a positive attitude toward the exercise system, enjoyed using the exercise system for exercises, and expressed their intention to continue using the exercise system. The interviews further uncovered the following 6 themes related to positive perceptions of the exercise system: (1) ease of use, (2) benefits, (3) enjoyment, (4) assistance, (5) accessibility, and (6) game design. Conversely, 3 themes were associated with negative perceptions: (1) difficulty in handling VR devices, (2) uncomfortable experiences when using VR devices, and (3) monotony. The interview findings may help interpret the results obtained from the questionnaire and provide further understanding of the factors influencing the patients’ acceptance and use of VR-based rehabilitation programs. For example, some patients expressed interest in the highly immersive game scenarios, which might have attracted them to use the exercise system to perform exercises. In contrast, some patients expressed undesirable feelings about wearing the headset, which might have led to a negative perception regarding the exercise system and impeded its use. Moreover, our findings serve as a concrete illustration of established technology acceptance models, such as the Technology Acceptance Model and Unified Theory of Acceptance and Use of Technology model. For example, previous research has underscored perceived usefulness as a key determinant of technology acceptance by users [23,24,32,33]. Our study further elucidated that motor function, daily living skills, cognitive ability, and mood were the main aspects of usefulness valued by the participants. In the following sections, we delve into the questionnaire and interview findings in greater detail.

#### Perception Ratings

In contrast to previous studies reporting that individuals with limited prior technology experience are less inclined to accept the technology [8,32,34-37], our findings indicated that a lack of experience with VR may not impede patients’ positive feelings about using the exercise system. The interview responses, which are discussed in the following sections, may help explain this. However, the highly positive perceptions might be a result of selection bias, meaning that participants who were satisfied with the exercise system were more likely to adhere to the VR exercises, whereas those who were unsatisfied with it could have withdrawn from the study. Therefore, the patients’ perceptions of the exercise system might have been overestimated.

#### Ease of Use

Ease of use is a critical perceptual aspect affecting technology acceptance [23,24,32,33]. Most patients who have experienced stroke are older adults who often have limited experience with technology and may face declining physical or cognitive functions, as well as technology anxiety [38-41]. Therefore, ensuring that VR-based rehabilitation exercises are easy to use is particularly important for this demographic to promote acceptance and adoption. In this study, almost all participants reported that it was easy to understand how to play the rehabilitation exercise games and use the VR devices. Although 1 participant initially found the games difficult due to unfamiliarity, the participant quickly adapted and found the games easier after practicing several times. This may be because the games were related to daily life (eg, fishing), and there were no complicated tasks or complex operations with the handheld controller. Consequently, participants could easily become familiar with the rules of the games and the use of the VR devices without much training or practice. Another possible explanation may be that most participants were able to perform the exercises independently. Necessary assistance was provided only to ensure their safety and prevent the development of abnormal movements (eg, shrugging the shoulders) by the researcher (JC), with the guidance of physical therapists [11]. These findings suggest several specific implications for VR-based rehabilitation programs. First, the simplicity of game design, mimicking daily life activities, can significantly enhance usability for patients with stroke. Second, minimal training and practice requirements make it feasible for older adults with limited technology experience to quickly adapt to the exercises. Third, providing tailored support to prevent incorrect movements can further improve patient engagement and safety. Consequently, designing technology-based rehabilitation exercises with these considerations can foster greater acceptance, enhance patient adherence, and ultimately improve rehabilitation outcomes for stroke survivors.

#### Benefits

Participants perceived improved upper limb motor function and daily living skills after using the exercise system. Although this exercise system was initially designed for motor rehabilitation, participants perceived improvements in reaction speed, thinking, and mood. Such gains have rarely been reported in previous studies. One possible explanation may be that the repetitive practice led to familiarity with the exercises, thereby helping the participants increase their reaction speed. Furthermore, the setup of the games in the exercise system, such as the 1-minute countdown and real-time feedback on task performance (refer to details of the games in Figures 1-5 and Multimedia Appendix 1), might provide participants with a sense of tension and competition. This, in turn, might “facilitate your thinking and make the brain work hard,” as 1 participant expressed. Another reason may be the advantages of immersive VR; that is, wearing the headset isolated the participants from the physical world, so they could fully focus on the tasks in the games. Moreover, being able to complete the tasks in the games provided the participants with a sense of achievement, which might have elicited positive emotions. As 1 participant described, being able to play games made the participants feel “useful,” and thus, “the feeling of pessimism” has been alleviated. Furthermore, the natural scenes and relaxing background music in the games might have helped relieve the participants’ negative emotions due to the stroke. A participant stated as follows:

I have been kind of stressed since I got this disease, but once I listen to that music, [my] mood turns good... walking into that wood and picking apples like [in] real [life], and being immersed into that environment. I feel like... my mind broadens.

The results indicate that the positive experiences associated with these games can help reduce the psychological burden of rehabilitation, making the process feel less like a chore and more like a recreational activity. These findings also suggest that the exercise system possesses the potential to influence multiple dimensions of stroke rehabilitation, which should be considered when designing and implementing such VR-based interventions.

#### Enjoyment

Consistent with previous studies that used video games for rehabilitation [42-46], performing exercises by playing the games in the exercise system was described as “exciting” and “fun” in this study. These findings suggest that the exercise system offers an enjoyable possibility for performing upper limb exercises. The enjoyment factor has specific implications for VR-based rehabilitation programs. First, enjoyable exercises can increase patient motivation and adherence and can consequently lead to more consistent participation in the rehabilitation regimen. This is particularly important for stroke survivors, as sustained engagement is crucial for recovery. Second, incorporating enjoyable elements into the exercise design can help alleviate technology anxiety, making patients more willing to try and continue using the system. Motivational and enjoyable elements can include reward systems that provide a sense of accomplishment, features that facilitate social interaction, and real-time positive feedback and encouragement.

#### Assistance

Some participants expressed that the virtual objects in the games guide and encourage them to perform movements that they might not be able to perform in the real world. For example, 1 participant said, “That apple-picking [game], together with that bird, could guide my hand to perform the movements.” The rationale behind this effect may be that learning a motor task is more effective when learners’ attention is focused on achieving specific goals (ie, focusing on external cues) rather than focusing on performing specific movements (ie, focusing internally on the movements themselves) [47-49]. For example, the apple-picking game was designed for wrist flexion and extension exercises. It required the participants to extend their wrists to pick a red apple and flex their wrists to drop it. Picking up and dropping the apple (ie, external focus) was relatively easy for the participants to understand because the instructions were specific and the goal was clear. However, if the participants were told to contract and relax muscles in the wrist and forearm (ie, internal focus), it would have been too abstract to understand and execute the movements. These findings suggest that the objects in virtual environments should be carefully designed because they play a vital role in helping patients learn movements and perform therapeutic tasks.

#### Accessibility

The participants reported that the exercise system provided extra opportunities to perform rehabilitation exercises, reflecting their need for exercises in addition to conventional rehabilitation therapy. A possible explanation may be that the needs for rehabilitation in patients who have experienced stroke, may not always be fulfilled because of constraints on professional and institutional resources in public hospitals [50,51]. For example, the patients in this study usually had to wait several days before getting an appointment with a physical therapist. The patients could only receive 30 minutes of physical therapy with the guidance of therapists every day during hospitalization [11], which was less than the treatment duration suggested in previous research [52,53].

#### Game Design

The games in the exercise system were characterized as “novel”; the virtual scenes and auditory components within the games offered the participants a highly immersive experience, described as “being in a wonderland.” These findings suggest that the virtual environment design and the VR device type might significantly impact user experience, which should be carefully considered when designing VR-based interventions. The implications of these findings are multifaceted. First, the novelty and immersive nature of the virtual environment can greatly enhance user engagement and enjoyment, making the rehabilitation process more appealing to patients. This implies that designers should focus on creating visually rich and interactive environments that capture patients' attention and interest. Second, the type of VR device used can affect the level of immersion and comfort. High-quality VR devices that offer clear visuals, realistic audio, and ergonomic design can prevent discomfort and enhance the overall experience. This suggests that investment in advanced VR hardware could be beneficial. Third, the positive descriptions of the experience indicate that carefully crafted virtual environments can provide a significant psychological boost to patients. Feeling as though they are “in a wonderland” can distract patients from the monotony of repetitive exercises, making the rehabilitation process feel less burdensome and more enjoyable. Fourth, these findings suggest that regular updates and new content should be incorporated to maintain novelty and engagement levels. Stale or repetitive content could undermine the benefits of the initial immersive experience, so ongoing development and refreshing content are crucial.

#### Difficulty in Handling VR Devices

Negative perceptions were mainly related to using the headset and handheld controller. Wearing the headset was heavy, making participants feel sweaty and stuffy, the cable used to connect the headset to the laptop sometimes limited the free movement of the participants’ heads, and the handheld controllers were heavy for some participants. This is due to the technical limitations of current VR technology. A large headset is necessary to support a high-resolution display, and a physical cable is needed to support the transmission of massive amounts of real-time data at a high speed and low latency. A better user experience with an immersive VR device relies on advances in hardware and software. For example, the development of retina displays can reduce the size of a headset and improve its wearable comfort, and advances in wireless transmission technology can facilitate the development of wireless VR devices to promote users’ freedom of movement in virtual environments [54,55].

#### Uncomfortable Experiences When Using VR Devices

A few participants experienced mild and short-lived discomfort with the VR exercise system, such as dizziness and eyestrain, as observed by the researcher. These findings indicate that the exercise system overall was well tolerated by the participants. Nevertheless, factors that may be associated with the side effects of immersive VR should be further identified and minimized in the design and development of immersive VR-based interventions for patients. The implications of these side effects are crucial for the safe implementation of VR-based rehabilitation programs. First, identifying the root causes of discomfort, such as prolonged use, visual strain from screen resolution, or motion sickness due to discrepancies in visual and vestibular input, is essential. Addressing these issues could involve optimizing session durations, improving screen technology, and ensuring smooth and realistic motion tracking. Second, providing clear guidelines and training for patients on how to use the VR system correctly can help minimize discomfort. Educating users on taking regular breaks, adjusting headset fit, and recognizing early signs of discomfort can enhance their overall experience. Third, personalizing the VR experience based on individual tolerance levels can further mitigate adverse effects. Implementing customizable settings that allow patients to adjust brightness, contrast, and motion sensitivity can help tailor the experience to their comfort. Fourth, continuous monitoring and feedback mechanisms can be integrated into the VR system to track patient responses in real time. This would allow for immediate adjustments and support, ensuring that the therapy remains both effective and comfortable.

#### Monotony

Although the upper limb exercises were designed as games, repetitive practice can become boring and monotonous for some individuals, potentially lowering their motivation to adhere to the exercise system [56]. This finding underscores the need for further exploration of strategies to sustain patients’ interest in VR-based interventions in the long term. Several strategies can be considered. First, incorporating a variety of game scenarios and levels can keep the exercises fresh and engaging, thereby preventing boredom. For instance, rotating through different themes, such as sports, adventure, and daily activities, can maintain patients' interest. Second, designing exercises with progressively challenging tasks can provide a sense of achievement and keep patients motivated. Third, personalizing exercise routines to match the individual's progress and preferences can make the sessions more enjoyable and relevant. Fourth, integrating social elements, such as multiplayer modes or community challenges, can enhance the fun aspect and encourage patients to stay committed to their rehabilitation.

### Implications for Practice

This study offers the following implications for practice. First, the immersive VR-based exercise system was initially accepted by patients because it offered increased exercise opportunities and was easy to use, enjoyable, and beneficial for motor rehabilitation. Thus, health care technology developers, public health decision makers, and health care providers can consider the possibility of integrating such VR-based interventions to complement conventional therapeutic exercises. The aforementioned characteristics should also be addressed to increase the likelihood of system acceptance. Second, therapeutic games can function as an appealing approach to encourage patients to engage in exercises. The preferences demonstrated by patients toward the games within the exercise system, including well-designed virtual environments and a high level of immersion, offer valuable insights for future refinement of VR-based exercise systems. Consequently, this can enhance the user experience and foster the acceptance of such systems. Third, considering patients may have limited prior technology experience, exercise systems must prioritize ease of use. To achieve this, integrating usability inspections and tests throughout the developmental phase is recommended. This approach ensures that the newly developed systems align with users’ needs and expectations, ultimately making them usable and useful [57-62]. Fourth, it is important to recognize that patients may encounter discomfort during VR exercises. Although this aspect was highlighted by patients only a few times, health care professionals must remain vigilant about the potential risks of VR technology in the use of VR-based exercise systems. During the design phase, the types of VR devices to be used should be carefully chosen or evaluated, considering the patients’ abilities. In addition, elements within virtual scenes that might induce discomfort, such as poor environmental illuminance and highly realistic graphics, should be avoided. During the implementation phase, health care providers should meticulously assess patients’ susceptibility to side effects related to VR-based therapy. Education can play a vital role in minimizing the risk of discomfort (eg, advising patients to avoid rapid head movements in virtual environments), and individualized therapy plans should be tailored to each patient’s unique needs and circumstances.

### Implications for Research

This study’s findings provide implications for future research. First, possible reasons for the participants’ withdrawal from the study should be further examined, as these may shed light on factors that impede patients’ acceptance of and adherence to the intervention. Such information would help improve the design and implementation of VR-based interventions, improve patients’ experiences with and acceptance of such interventions, and increase their benefits for patients. Further studies are warranted to collect information using approaches such as interviews with patients and heuristic evaluation by experts [63]. Second, fewer statements related to negative perceptions of the exercise system were identified than those related to positive perceptions. One possibility for this finding may be that the respondents were inclined to be agreeable and positive to avoid the disapprobation of the interviewers [64,65]. Future studies should consider the impact of response bias on the validity of the results, detect such biases when administering questionnaires and conducting interviews, and deal with the bias during data analysis. Third, the participants used the immersive VR-based exercise system for up to 2 weeks. However, poststroke motor rehabilitation is often a long-term process, and patients’ experiences with the exercise system may change with time. Further research is needed to examine patients’ perceptions of such VR-based interventions in the long term. For example, it has been suggested that >15 hours of total intervention are needed to produce improvements in upper limb motor function [66]. Further studies should accumulate patients’ experiences with such VR-based interventions at different time points. Fourth, the implementation of VR-based interventions requires the involvement of health care providers and caregivers, whose attitude toward such interventions can also influence patients’ acceptance of and adherence to VR-based therapy [24,32,33]. Therefore, research examining the opinions of health care providers and caregivers regarding VR-based interventions is warranted.

### Limitations

This study has several limitations. First, the participants included in the study were predominantly male individuals (15/21, 71%), were in the subacute stage of stroke (≤6 months after stroke onset), and had limited experience using VR devices. Therefore, further research is needed to assess the generalizability of the findings to other user groups. Second, some withdrawals from the study may have been caused by dissatisfaction with the VR-based exercise system, which might have led to an overestimation of the participants’ perceptions. In addition, the participants’ reasons for withdrawal were not recorded, resulting in their barriers to accepting and adhering to VR exercises and the usability issues of the exercise system being unknown. Third, participants’ perceptions of the exercise system were based on a relatively short period of use. Long-term perceptions of the system should be investigated in future studies. Fourth, some interviews were brief. Consistent with challenges documented in relevant studies [67,68], some of our participants exhibited limited communication and articulation. This limitation could be attributed to reduced sensitivity to perceptions and increased susceptibility to fatigue following brain injuries. For future studies, it may be beneficial to explore other strategies that could improve overall understanding, such as engaging caregivers and family members who can represent patients’ experiences to provide complementary information; using group discussions to enhance participation, expression, and recall; and establishing user-friendly communication methods (eg, avoiding highly technical terms) [67-69].

### Conclusions

Integrating VR to support poststroke functioning improvement exercises appears promising and acceptable based on patient interests. However, individual preferences and adaptability must be considered to achieve optimal outcomes, as some patients may find VR challenging. VR-guided exercise approaches of this nature should be simple to use, promote improvement in health conditions, foster a sense of fun, facilitate exercise access, and be well-designed in terms of VR exercise content. In addition, these approaches must address challenges such as bulky VR equipment, motion sickness, discomfort, and exercise monotony. Overall, assessing and addressing both favorable and unfavorable patient concerns should be done early in the development process, as this is critical for diffusion and adoption.

## Appendix

Multimedia Appendix 1 Games developed and used by the intervention group in the randomized controlled trial.

Multimedia Appendix 2 Commercial games used by the control group in the randomized controlled trial.

## Abbreviations

RCT: randomized controlled trial
VR: virtual reality

## Data Availability

The datasets generated during and analyzed during this study are available from the corresponding author upon reasonable request.
