# Supplementary material for: Perceptions of Patients With Stroke Regarding an Immersive Virtual Reality–Based Exercise System for Upper Limb Rehabilitation: Questionnaire and Interview Study
Source: JMIR Serious Games. 2025 Jan 1;13:e49847. doi: 10.2196/49847 (PMC11736226; doi:10.2196/49847)
Supplement: Multimedia Appendix 1 [file games_v13i1e49847_app1.docx]

**Appendix 1. Games developed and used by the intervention group in the RCT.**

***Dumbbell-lifting game****.*  This game included two modes: shoulder flexion and shoulder abduction exercises. The participants held the controller (presented as a dumbbell in the VR environment) and needed to (1) start with their arm hanging naturally to the side of their body, elbow fully extended, and palm facing the side of the body; (2) lift the controller up and out to the front (for flexion) or to the side (for abduction) until it reached shoulder level, keeping the elbow straight; and (3) hold the controller at the shoulder level for 1−3 seconds, depending on the difficulty level. A longer time required for holding the controller indicated a higher difficulty level. One trial lasted 1 minute, during which each participant was required to repeatedly lift the controller by following the above-described steps as many times as possible. For each mode, four trials were performed by each participant.

***Fishing game****.* This game was designed as an elbow flexion exercise. The participants were placed in front of a pond in the VR environment in which fish were swimming. The participants needed to (1) hold the controller (presented as a fishing rod in the VR environment) and wait until it started vibrating, indicating that a fish had been caught; (2) flex their elbow and raise the controller toward the shoulder, keeping the elbow still on the real table; and (3) hold the position for 1−3 seconds, depending on the difficulty level, to pull the fish out of the water. A longer time required for holding the controller indicated a higher difficulty level. One trial lasted 1 minute, during which each participant was required to catch as many fish as possible. Each participant performed four trials.

***Sheep-whacking game****.* This game was designed as forearm pronation and supination exercises. The participants were placed in front of two holes in the VR environment, holding the controller (presented as a hammer in the VR environment). A sheep alternately popped up from one of the two holes. The participants needed to whack the sheep back into the hole by pronating or supinating their forearm, with the elbow kept still on a real table. There were four difficulty levels, in which the participants needed to pronate and supinate their forearm to 15°, 30°, 45°, and 60°. The greater degree of movement required indicated a higher difficulty level. One trial lasted 1 minute, during which each participant was required to whack as many sheep as possible. Each participant performed four trials.

***Apple-picking game****.* This game was designed as a wrist flexion and extension exercise. The participants were placed in front of an apple tree and a stump in the VR environment. The participants were asked to hold the controller (presented as a bird in the VR environment) and extend their wrist to control the upward flight of the bird to pick a red apple from the tree, then flex their wrist to control the downward flight of the bird and drop the apple on the stump. Immediately, another apple appeared on the tree, and the participants needed to repeat the process. There were four difficulty levels, in which the participants needed to flex and extend their wrist to 15°, 30°, 45°, and 60°. A greater degree of movement required indicated a higher difficulty level. One trial lasted 1 minute, during which each participant was required to pick as many apples as possible. Each participant performed four trials.

***Balloon-popping game***. This game was designed as a reaching exercise for the complete upper limb and combined the movements of shoulder flexion and extension and elbow flexion and extension. The participants were placed in front of a virtual table, on which a balloon appeared on the participant’s affected side. The participants needed to hold the controller (presented as a hand in the VR environment) and reach their hand toward the balloon and pop it by pressing a trigger button on the controller using their index finger. Immediately, another balloon appeared, and the participants needed to repeat the process. The game had four modes: reaching toward the front, reaching toward the side at 45° and 90°, and reaching upward (toward a balloon). There were four difficulty levels within each mode, depending upon the distance between the balloons and participants. Balloons randomly appeared within a distance of 0%–25%, 0%–50%, 0%–75%, and 0%–100% of the participants’ maximum reaching distance. A longer distance indicated a higher difficulty level. Each participant’s maximum reaching distance was measured before the game. One trial lasted 1 minute, during which each participant was required to pop as many balloons as possible. Each participant performed four trials.
