# Supplementary material for: Perceptions of Patients With Stroke Regarding an Immersive Virtual Reality–Based Exercise System for Upper Limb Rehabilitation: Questionnaire and Interview Study
Source: JMIR Serious Games. 2025 Jan 1;13:e49847. doi: 10.2196/49847 (PMC11736226; doi:10.2196/49847)
Supplement: Multimedia Appendix 2 [file games_v13i1e49847_app2.docx]

**Appendix 2. Commercial games used by the control group in the RCT.**

***Chocolat Rush****.* Chocolat Rush is a shooting game. The participants stood in the center of a room in the virtual environment. They needed to hold the controller (presented as a gun in the VR environment) and press a trigger button on it using their index finger to fire bullets at monsters swarming in from different directions and blow them up. The participants achieved higher scores when they hit more monsters. This game had no time limit.

***Zooma****.* The participants stood on a high platform in a hall in the virtual environment. A string of balls of different colors was rolling in front of the participants. They needed to hold the controller (presented as a gun in the VR environment) and shoot different colored balls. The participants needed to eliminate all of the rolling balls by lining up three or more balls of the same color. The participants achieved higher scores when they eliminated more balls. Each trial lasted approximately 2 minutes.

***Fruit Ninja VR****.* Fruits popped up from the ground in front of the participants in the virtual environment. The participants needed to hold the controller (presented as a sword in the VR environment) and cut the fruits into pieces. The participants achieved higher scores when they cut more fruit. Each trial lasted approximately 1.5 minutes.

***Kooring Wonderland – Mecadino’s Attack game package****.* Two games in this package were used. (1) Mirrorland’s Saber: The participants needed to cut blocks flying toward them into two pieces using the controller (presented as a red or blue saber in the VR environment). The color of the blocks needed to match the color of the saber. (2) Mock Turtle’s Magic Shield: The participants needed to block arrows flying toward them with the controller (presented as a red or green shield in the VR environment). The color of the arrows needed to match the color of the shield. The participants won a gold, silver, or bronze medal, depending on the number of blocks they cut or arrows they blocked successfully. Each trial lasted approximately 2.5 minutes.

***Kooring Wonderland – Heart Castle Crush game package****.* Three games in this package were used. (1) Humpty Dumpty: The participants needed to hold the controller (presented as a red or blue hammer in the VR environment) and whack the head of Humpty Dumpty running closer to them in the virtual environment. Humpty Dumpty disappeared immediately after being whacked successfully. The color of Humpty Dumpty needed to match the color of the hammer. (2) Garden to Live Flowers: The participants stood in front of a tree in the virtual environment. The participants needed to hold the controller (presented as a paintbrush in the VR environment) and paint the white flowers on the tree red. The painted flowers disappeared immediately, and white flowers kept showing up. (3) Koorobo Toorobo: There were four buttons of different colors (purple, green, yellow, and red) in front of the participants in the virtual environment, and different colored blocks kept showing up in a circle in the distance. The participants needed to hold the controller (presented as a virtual hand in the VR environment) and press the corresponding button according to the color of the block. The participants won a gold, silver, or bronze medal, depending on the number of Humpty Dumpty’s they hit, flowers they painted, or buttons they pressed successfully. Each trial lasted approximately 2.5 minutes.

***Space Slurpies****.* Food (in the shape of a cube, tetrahedron, icosahedron, etc.) was floating in the air and moving around the participants in the virtual environment. The participants needed to hold and move the controller (presented as a snake in the VR environment) to keep eating the food, during which the snake grew in length and size. The participants achieved higher scores when the virtual snake ate more food. Each trial lasted approximately 2 minutes.
